# Development and initial evaluation of a rigid rhinoscopy model as a pedagogic tool in veterinary medicine

**DOI:** 10.3389/fvets.2024.1356026

**Published:** 2024-09-10

**Authors:** Bethany Sabatino Myerow, Jessica C. Pritchard, Kathryn Kalscheur, Steve Marks, Kenneth Royal, Nicholas Thoreson, Noah Pollard, Eleanor C. Hawkins

**Affiliations:** ^1^Veterinary Medical Center, College of Veterinary Medicine, University of Tennessee, Knoxville, TN, United States; ^2^School of Veterinary Medicine, College of Engineering, University of Wisconsin-Madison, Madison, WI, United States; ^3^College of Veterinary Medicine, Clemson University, Clemson, SC, United States; ^4^College of Veterinary Medicine, North Carolina State University, Raleigh, NC, United States

**Keywords:** rhinoscopy, model, simulation, pedagogic, training, tool

## Abstract

No model exists to train the handling skills required for successful performance of rigid rhinoscopy in veterinary patients. The complex anatomy of the nasal cavity, the limited space between turbinates, and the propensity of the mucosa to bleed with slight trauma make thorough examination of a nasal cavity difficult. The goal of this study was development and initial testing of a low fidelity canine rigid rhinoscopy training model for veterinary novices. A model comprising three tubes of various lumen diameters that were connected to a conduction system was created. Each tube contained three colored stars and a number that were to be described by trainees. The model both counted contacts and audibly beeped if pressure was applied by the rhinoscope to the sides of the tube. Board-certified veterinary internists (experts) and veterinary students (novices) performed two timed simulation exercises during a single session. Participants completed a questionnaire before and after the simulations to assess model utility. There were no statistically significant differences in contacts or time to completion between novices and experts. Novices provided feedback that the model improved their ability to control the rhinoscope, helped them learn to perform rigid rhinoscopy, was enjoyable, and was appropriately challenging. Expert feedback included that the model was a potentially useful pedagogic tool for training rigid rhinoscopy, including hand control and indirect hand-eye coordination, and was appropriately challenging. We conclude that this rhinoscopy model has potential to be an effective teaching tool for novice rhinoscopists. With minor modifications, the model could provide additional challenges.

## Introduction

Nasal disease is a common cause for referral to veterinary specialists, and well-developed rhinoscopy skills are essential for optimal patient outcome. The treatment of choice for nasal foreign bodies is rhinoscopic retrieval. For dogs with nasal discharge, masses, or congestion, definitive diagnosis is best accomplished with rhinoscopy and biopsy in combination with computed tomographic imaging (1, 2). Rhinoscopy combined with biopsy has a reported diagnostic success rate of 83–90% (3, 4). However, rhinoscopy is a difficult technique to master, and the success of rhinoscopy for a given patient is dependent on operator ability. Nasal anatomy is composed of the dorsal, middle, and ventral meatus (with the nasal septum separating the right and the left nasal passages). Successful rhinoscopy requires visualizing the nasal passages on a video screen while executing steady movements of a rigid scope between the nasal turbinates to minimize trauma and bleeding. Furthermore, nasal pathology (e.g., swollen mucosa, increased tissue friability, profuse mucus or exudate, pre-existing hemorrhage, distortion of normal anatomy, masses, etc.) can obstruct visualization and increase the risk of trauma. In addition, most patients require the concurrent manipulation of forceps, brushes, catheters, or lasers to obtain diagnostic samples or perform intricate corrective procedures (5, 6).

Novice rhinoscopists, currently often internal medicine residents, have limited means to master the aforementioned skills. Learning on client-owned animals is most common and has several disadvantages for the trainee and patient including increased anesthesia time, incomplete exams, increased nasal trauma, and trainee lack of procedural confidence (7). While cadavers or purpose-bred live animals provide the benefit of realism, training opportunities are sparse, often occur in a single session, and are insufficient to allow for the development of proficiency in the trained skills (8, 9). Additionally, there are growing concerns with the use of purpose-bred animals for training of veterinary students and residents (10).

In human medical education, the use of high and low-fidelity procedural simulators is common and numerous studies demonstrate their efficiency and adequacy for skills training, and their cost-effectiveness (8, 9, 11–16). Similar findings have been demonstrated in veterinary medicine for flexible endoscopy training (17). Given the current deficiencies in rhinoscopy training, there exists a critical need for a low fidelity rhinoscopy model to optimize trainee learning. Such a model would facilitate the development of scope handling skills, including hand control, indirect hand-eye coordination during monitor viewing, and the manipulation of biopsy and retriever forceps. Moreover, use of effective models can improve animal welfare by limiting the use of purpose-bred animals for training and improving efficiency in novice endoscopists. Thus, the goal of this study was the development and initial evaluation of a rigid rhinoscopy model as a pedagogic tool in veterinary medicine. We hypothesized that a useful model could be created that would distinguish between experts and novice users in number of mucosal contacts and time to exam completion. We hoped the model would be useful as an initial tool in the acquisition of skills relevant to rigid rhinoscopy.

## Materials and methods

The North Carolina State University Institutional Review Board (IRB) deemed this study exempt.

### Development of the rhinoscopy model

The rhinoscopy model is in the process of development by UW-Madison undergraduate biomedical engineering students (NT and NP) under the supervision of an engineer with expertise in medical applications (KK). The goal was to develop cavities that would allow the passage of a rigid rhinoscope and forceps with the following features: allowing some contact against the wall of the cavity but only to the extent that trauma would be avoided in a live patient; incorporating gamification aspects to encourage habitually performing a complete examination, to encourage consistent practice; and to minimize cost. The emphasis was on the development of hand-eye-screen coordination to guide the scope into relevant regions.

**Figure 1:**
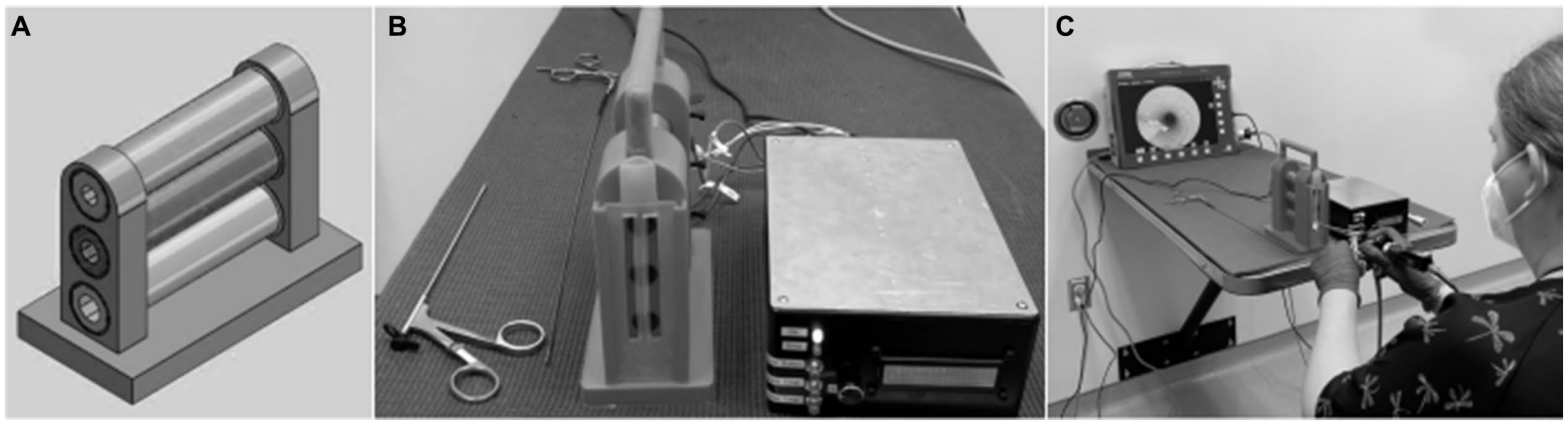
Design schematic of frame with three vertically-stacked tubes of varying lumen size in place **(A)**. A vertical rubber strip was placed on the outside of the tubes to create the feeling of the alar fold. The final product of rhinoscopy simulator comprised three stacked tubes each connected to the control box **(B)**. An investigator (BSM) demonstrated use of a rigid rhinoscope to evaluate the tubes of the training model and to remove a star sticker from one of the tubes **(C)**.

The model has a small lightweight frame of 3D printed polyactide (PLA) plastic with three vertically stacked straight tubes with a modular and angled design (Figure 1A). The inside of the tubes is lined with a pressure sensitive resistor, followed by a 3D printed flexible elastic resin. An audible signal (“beep”) alerts the user when pressure is being applied inside of the tubes. The pressure limit was set by one of the authors who regularly performs rigid rhinoscopy working with the engineers to demonstrate what degree of pressure might be acceptable.

Wires connect each tube to a control box (Figure 1B). The control box contains a simple Arduino Uno ® (Italy) and breadboard. The sensitivity is controlled through a knob on the outside of the control box. In addition to the audible signal that occurs, each contact is cumulatively counted and displayed on an LCD screen. A reset button allows for zeroing of the counter. To increase trainee interest, and to allow investigators to confirm thorough visualization by participants during this study, three small, adhesive stars of different colors were adhered at different locations within each tube, and an adhesive number was positioned at the far end of each tube. To further increase interest, the most distal star in the ventral tube was not adhered in place. The star could be removed and replaced to allow for practice of forceps handling with rhinoscopic guidance. Details for the construction of the model are provided in Appendix 1. The approximate cost for materials to construct the model was $416.

### Study groups

Fourth year North Carolina State University College of Veterinary Medicine students (novice group) voluntarily self-enrolled by responding to fliers and emails. The expert group was comprised of board-certified veterinary internists, who regularly performed or trained rigid rhinoscopy over the past 3 years in the local area and responded to individual emails. Experts were from private practice and university veterinary hospitals. Based on our convenience sample of experts, we aimed to recruit twice as many novices as experts as has been done for similar studies (18). Novices were excluded if they had any prior *hands-on* experience with any rigid or flexible endoscope. Experts were excluded if they had not performed or trained rigid rhinoscopy within the last 3 years.

### Simulation sessions

Each participant met individually in a closed room with one of the authors (BSM) for a single session during which a pre-simulation questionnaire was completed, two simulated rhinoscopies were performed, and a post-simulation questionnaire was completed. Immediately after completion of the pre-simulation questionnaire, BSM read the participant a script that described the model and the goals of the simulations. The participant was told that the entire process of completing both simulations would likely be less than 15 min, but that up to 30 min would be available. The post-simulation questionnaire was completed immediately following completion of both simulations.

A zero degree 2.7 mm rigid video rhinoscope was used for all simulations (Hopkins II Straight Forward Telescope 0°, Karl Storz Endoskope). The light intensity of the scope and the pressure sensitivity of the model remained constant throughout the study. The goal of each simulation was for the participant to visualize the full length of each tube while minimizing contact with the sides of the tube. To confirm that the participant was visualizing the full length of the tube, the participant was asked to state the color of each star and the value of the number at the end of the tube as they were encountered.

For the first simulation, the participant began with the largest diameter (dorsal) tube. A scope cover was used (16 cm × 8 mm protective cover) to widen the diameter of the rhinoscope to make evaluation of this tube more challenging. Participants then removed the cover and examined the middle and ventral tubes. After describing the stars and number in the ventral tube, they chose either flexible or rigid forceps and used them to remove the most distal star from the tube (Figure 1C). The forceps were inserted next to the unsheathed scope to remove the star. If the participant had difficulty with the first forceps selected, they were permitted to attempt the task with the other. For the second simulation, the procedures were repeated with the exception that the participants replaced the star in the ventral tube using forceps alongside the scope.

Performance was assessed by the number of contacts recorded on the control box. Contacts were recorded for each tube examined and for each manipulation of the star. During some simulations, the model did not “perceive” the correction of a single contact between the scope and the lining of the tube, and it continued to record multiple, erroneous contacts in rapid succession. When this occurred, BSM recorded the number of contacts prior to the error, and reset the counter. For data recording these events were noted as “infinity contacts” and given a value of one contact. Time for each simulation was measured with a stopwatch, beginning with the insertion of the scope into the dorsal tube and ending when the scope and forceps were fully withdrawn from the ventral tube following manipulation of the star.

### Questionnaires

Pre-simulation questionnaires for both novices and experts included participants’ age, and their self-assessment of hand-eye-screen coordination (1–5; 5 excellent). The novice pre-simulation questionnaire also included the number of endoscopy procedures the participant had previously observed (none, 1–2, 3–10, 11–25, or >25). The expert pre-simulation questionnaire included a question about the viewing angle of the rhinoscope the participant typically uses. The experts were also asked to provide their assessment of the difficulty of performing rigid rhinoscopy and of the difficulty of training rigid rhinoscopy (1–10; 10 extremely difficult). Free text boxes were included for elaboration of the particular aspects of performing and training rigid rhinoscopy the experts find particularly challenging.

Post-simulation questionnaires focused on the participant’s assessment of the model from a trainee perspective (novice questionnaire; Appendix 2) or instructor perspective (expert questionnaire; Appendix 3). For both questionnaires, statements were made regarding the model and its usefulness, accompanied by a 4-point Likert-type scale (1-strongly disagree to 4-strongly agree). Comment boxes were included for all participants to provide feedback regarding positive and negative aspects of the model and their experience with it.

After data collection, a thematic analysis of open-ended responses was performed by BSM, ECH, and JCP. Responses were categorized as positive, negative, or neutral. Positive responses were further grouped as related to: the realism of the model; the experience of handling the scope or instruments, or hand-eye-screen coordination; the feedback provided by the beep; the experience of the star manipulation task; the low stress experienced; and, a recommendation that no changes be made. Negative responses were further grouped as related to: stress resulting from the beep; and, need for more challenging options.

### Data analysis

All statistical analyses were performed using commercially available software (SigmaPlot 14.0, Inpixon, Palo Alto, CA 94303). Data are presented as median values (25th, 75th percentile). Mann–Whitney *U* tests were used to compare data between novices and experts. Wilcoxon signed rank tests were used to compare paired data within groups. Potential relationships between number of contacts and elapsed time, and between age of participant and number of contacts, were investigated using Spearman’s rho correlation coefficients. The level of significance for all statistical analyses was *p <* 0.05.

## Results

### Pre-simulation questionnaire responses

A total of 32 novice and 16 expert participants were enrolled. Novices were younger [26 (25, 27) years] than experts [40 (35, 58) years; *p* < 0.001] and had lower self-assessment scores for hand-eye-screen coordination [2 (3, 4), with 5 being excellent] compared with experts [4 (4, 4); *p* < 0.001].

Novices had rarely observed flexible or rigid endoscopy. Four (12.5%) of the novices had never observed endoscopy, while 26 (81.3%) participants had observed 1–10 procedures, and 2 (6%) had observed 11–25 procedures. Experts most often had experience using a 0-degree rhinoscope, either solely or along with 5- and/or 30-degree scopes (*n* = 10, 62.5%). The remainder (*n* = 6; 37.5%) used either a 5-degree or 30-degree scope or were uncertain what angle scope they used.

Experts considered rhinoscopy to be moderately difficult to perform [4.5 (3, 7), with 10 being extremely difficult], and to train [5 (4, 7.8)]. Aspects of rhinoscopy considered to be challenging by experts included: iatrogenic hemorrhage, small anatomical spaces, excessive mucus, patient movement, limitations of thorough exam, and biopsy of small lesions.

### Comparison of performance between groups (novices and experts)

**Figure 2:**
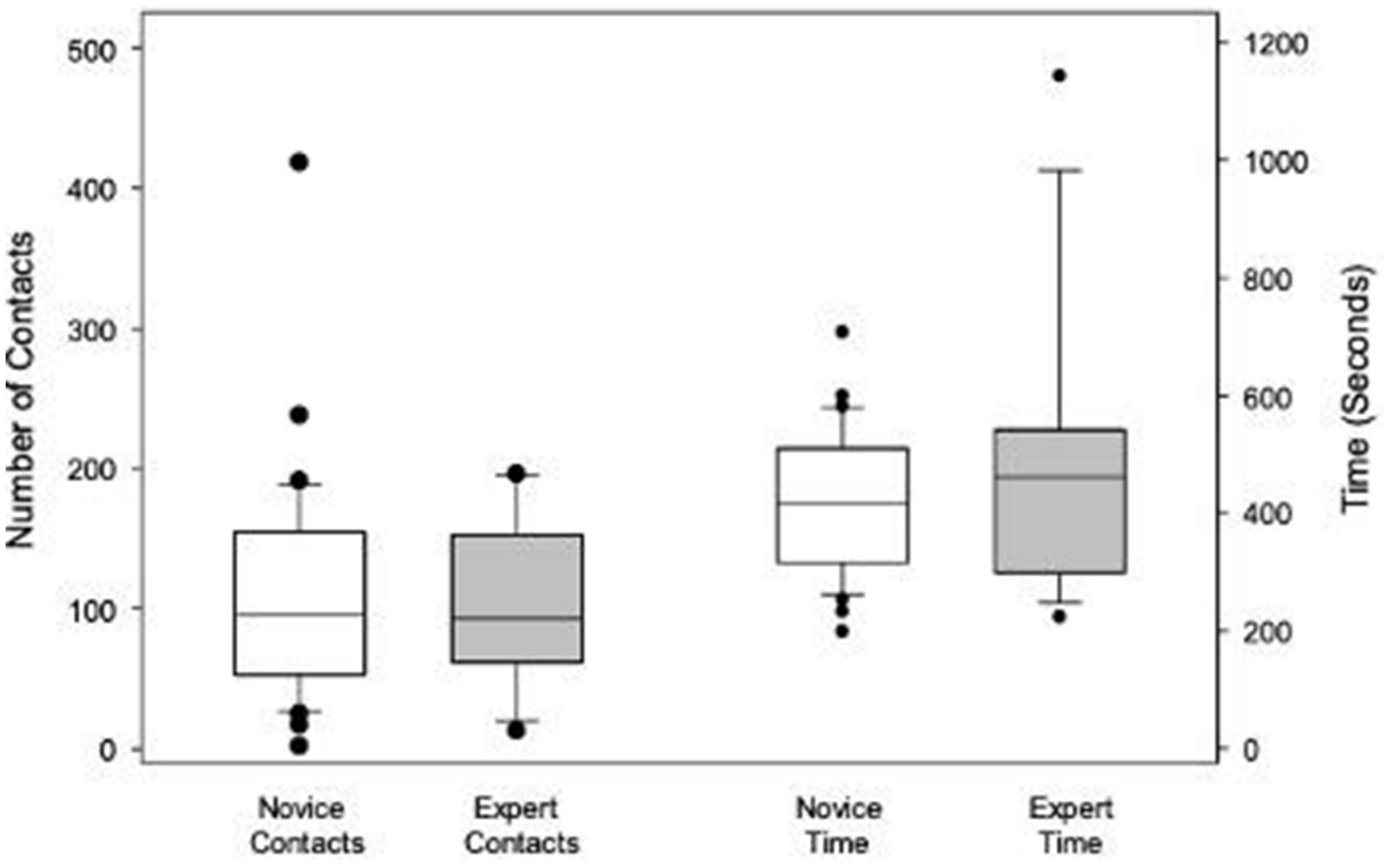
Box plots illustrating and comparing the total contacts and total elapsed time for both simulations combined for novices and experts. Boxes indicate the 25th, 50th, and 75th percentiles. Whiskers indicate the 10th and 90th percentiles. Dots denote outliers.

**Figure 3:**
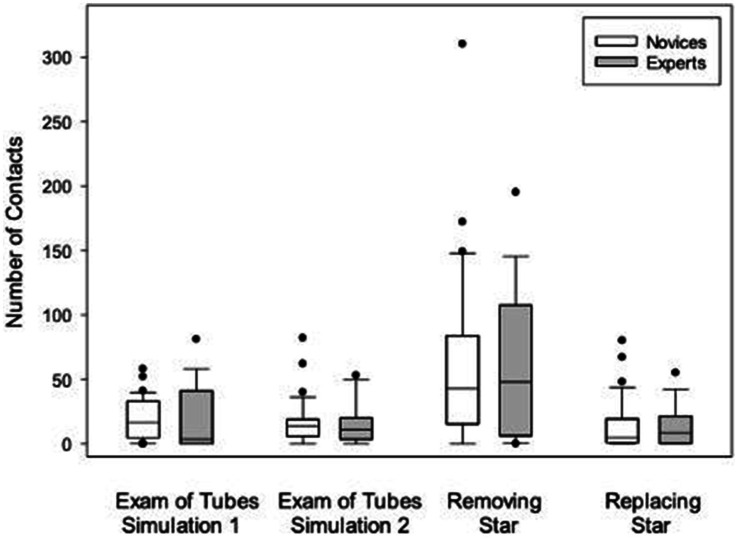
Box plots illustrating and comparing total number of contacts for novices and experts in each simulation, and removal, and replacement of the star. Boxes indicate the 25th, 50th, and 75th percentiles. Whiskers indicate the 10th and 90th percentiles. Dots denote outliers.

No differences were found between novices and experts for total contacts or elapsed time (Figure 2). Total contacts made during completion of both simulations were 96.0 (53.0, 154.8) for novices and 93.0 (61.8, 153.8) for experts (*p* = 0.965). Elapsed times for completion of both simulations were not different between novices and experts (*p* = 0.491). Similarly, no differences were found between groups for number of contacts when the examination of the tubes and the tasks related to the star were analyzed separately (Figure 3).

### Comparison of performance between first and second simulations within groups

Novices and experts had significantly more contacts during the first simulation compared with the second (*p* < 0.001 and *p* = 0.044, respectively), but this difference was no longer present when the tasks of removing the star (first simulation) and replacing the star (second simulation) were excluded (Figure 3). Novices had significantly more contacts removing the star compared with replacing it (*p* < 0.001). Similarly, experts had significantly more contacts removing the star compared replacing with it (*p* = 0.013). When the tasks of removing or replacing the stars were removed from analysis, no differences between simulations were found for either novices or experts (*p* = 0.903). Novices had 16.5 (4.8, 33.0) contacts during the first simulation, compared with 14.0 (6.0, 19.0) during the second (*p* = 0.330). Experts had 3.5 (0.0, 41.0) contacts during the first simulation, compared with 11.0 (3.5, 20.0) during the second.

Similarly, the time to complete the first simulation was longer than the second for both novices (*p* < 0.001) and experts (*p* < 0.001). Total times and contacts for both simulations were not different between novices and experts (*p* = 0.491 and *p* = 0.965, respectively; Figure 2). It was not possible to objectively investigate whether these differences were a result of the different level of difficulty in removing compared with replacing the star, because times were not recorded for each step of the simulations.

### Performance relative to pre-simulation questionnaire responses

Age was not associated with number of contacts for either novices (*p* = 0.604), experts (*p* = 0.336) or both groups combined (*p* = 0.881). There was no relationship between the number of contacts made by novices and their prior experience observing endoscopies, or their self-assessment of hand-eye-screen coordination. Nor was there a relationship between the number of contacts made by experts and their prior experience with a zero-degree scope, or their self-assessment of hand-eye-screen coordination. However, the number of participants within the sub-categories was often small.

### Post-simulation questionnaire responses

Novices were uniformly positive in their assessment of the model (Table 1). All novices agreed that practicing with the model would improve their ability to control the scope and would improve their ability to perform rigid rhinoscopy, with greater than 80% strongly agreeing. Similarly, all novices subjectively agreed that the model was enjoyable and an appropriately challenging way to promote growth of skills applicable to rigid rhinoscopy, with greater than 80% strongly agreeing. Only 9.4% of novices noted that the tasks were frustrating; the majority of novices (90.6%) disagreed or strongly disagreed with that statement.

**Table 1:** Post-simulation questionnaire results from novice and expert participants.

Novice (*n* = 32) statements	Strongly disagree	Disagree	Agree	Strongly agree
Practicing with this model would improve my ability to control the scope in rigid rhinoscopies	0	0	5	27
Practicing with this model would improve my ability to perform rigid rhinoscopies	0	0	6	26
The use of this model is an appropriately challenging way to promote growth of my skills applicable to rigid rhinoscopy	0	0	5	27
Performance of these tasks was an enjoyable way to practice skills applicable to rigid rhinoscopy	0	0	5	27
Performance of these tasks was a frustrating way to practice skills applicable to rigid rhinoscopy	10	19	3	0

Expert (*n* = 16) statements	Strongly disagree	Disagree	Agree	Strongly agree
The use of this model is an effective way to train skills applicable to rigid rhinoscopy	0	1	10	5
This model provides training appropriate for the performance of rigid rhinoscopy with respect to hand control	0	0	10	6
This model provides training appropriate for the performance of rigid rhinoscopy with respect to indirect hand-eye coordination	0	0	10	6
The use of this model is an appropriately challenging way to promote growth of skills applicable to rigid rhinoscopy	0	1	11	4
The use of this model is an enjoyable way to train skills applicable to rigid rhinoscopy	0	0	12	4
The use of this model is a frustrating way to train skills applicable to rigid rhinoscopy	5	7	3	1

Nearly all experts (93.8%) agreed the model was an effective way to train skills and was an appropriately challenging way to promote growth of skills applicable to rigid rhinoscopy (Table 1). All the experts agreed that the model provided appropriate training with respect to hand control and indirect hand-eye coordination. While all experts agreed that the model was an enjoyable way to train skills applicable to rigid rhinoscopy, four experts (25%) also indicated that the model was a frustrating way to train skills applicable to rigid rhinoscopy.

### Open-ended response feedback

All experts and novices provided comments regarding aspects of the model they found applicable to training novices in rhinoscopy. Two-thirds of participants (*n* = 32) commented on its utility in training scope or instrument handling, and hand-eye-screen coordination. Half of experts (*n* = 8) specifically listed “hand-eye coordination” as an aspect of the model they found useful. Nine participants (19%) noted that the beep resulting from contact was helpful for immediate feedback. Two participants (4.2%), one novice and one expert, commented that the model was “low stress.” Other comments focused on the realism of the model (*n* = 7, 15%), and manipulation of the star (*n* = 8, 15%).

All participants also provided comments regarding aspects of the model they would change. Eleven participants (23%), all of whom were novices, noted they would not change anything about the model. In contrast to responses to the previous question, twelve participants (25%) wrote that the beep was inaccurate or stressful. Fifteen participants (31%), a mixture of experts and novices, suggested making the model more complex or challenging, including adding more places to explore, more obstacles, or more foreign objects.

Most novice participants (*n* = 23, 72%) and expert participants (*n* = 10, 69%) responded to the option to provide additional feedback. The most common theme was a supportive statement about enjoyment while using the model or a desire to perform the tasks again (13 novices, 6 experts, 58%). Novice participants noted “I had fun participating—I want to practice again!” and “I really enjoyed the opportunity for hands-on practice—this seems like a great teaching tool!” While experts commented “This is a clever way to train, and I enjoyed using it. I think lots of potential!” and “Very fun!” Remaining comments focused on like or dislike of the beep (12%), suggestions to make the model more challenging (6%), positive utility with scope handling or hand-eye-screen coordination (6%), that no improvements were needed (6%), or miscellaneous suggestions related to the simulation (12%).

## Discussion

This study describes the development of and initial evaluation of a rigid rhinoscopy model as a pedagogic tool in veterinary medicine. This low-fidelity model was relatively simple, lightweight, transportable, and inexpensive to make. Post-simulation questionnaires for both novices and experts were strongly supportive of the use of the model for the development of hand-eye-screen skills applicable in rigid rhinoscopy.

No differences were found between the performance of novices and experts using the model.

While this could be the result of a design flaw of the model, such as lacking appropriate difficulty or excess sensitivity of the model to scope touches, factors related to the study design provide alternative explanations. It is possible that experts would have performed better using a rhinoscope of their choice. Student stress was specifically considered in the study design, and BSM’s role as a teaching fellow within the context of a private, non-classroom environment offered students a low-stakes environment. Experts may have found it stressful to meet self-imposed expectations when performing a clinical task in front of another specialist.

Although our exclusion and inclusion criteria were designed to appropriately distinguish experts from novices, some participants may have been miscategorized. Many of the experts were faculty in academic practices who primarily trained others in rigid rhinoscopy rather than regularly directly performing rhinoscopy themselves. We did not ask the experts to quantify the number of rhinoscopies that they had performed or trained. The timing of the last rhinoscopy that the experts had performed was also not assessed. This study was not designed to assess the durability of rhinoscopy skills, and the longevity of endoscopic skills is largely unknown (19). Additionally, we did not assess novice or expert experience in other realms involving hand-eye-screen coordination such as robotics work, plumbing, juggling, darts, and wood working. Little data exists to show how these skills may transfer to endoscopy.

Novices and experts were both familiarized to the stimulations in the same manner (a written script was read to them prior to the simulations). The novices may have learned enough from the familiarization to be at a higher level of knowledge than they would have been without it. Another possible explanation for the high performance of novices, comparable to experts, is their young age and presumed long-term prior exposure to video games and computer screens (20). Median participant age for novices was 14 years younger than that of experts. An age-related effect could not be confirmed by our study.

We also did not see a statistical difference between the first and second simulations for either novices or experts. The purpose of the study was to develop and provide an initial evaluation of a rigid rhinoscopy model as a pedagogic tool in veterinary medicine. Only two simulations were performed, each was a relatively short duration. The second simulation was performed immediately following the first. Such a design would not be expected to have a strong training effect (21). It is possible that a difference would have been seen with more time and repetition, as this study may have been underpowered to detect a difference if one existed between the groups. A learning curve was not assessed because the first and second simulations were not identical (remove star vs. replace star). This would be an important point to be assessed in future studies that utilize repetition of the same task.

Both novices and experts commented in the open-ended responses that the model was useful for training scope handling and hand-eye coordination, two of our goals in model creation for training rigid rhinoscopy. While some respondents commented that the beep added to their stress or they preferred not to hear the beep, we feel the beep is important for immediate feedback so that participants can keep their eyes on the screen (21). Some participants suggested the model should have more challenging options. Since the tubes in the model are easily interchangeable, the creation of more complex tubes, such as those with a slight curve more closely resembling canine or feline nasal anatomy, would be a possibility as trainees progress. Importantly, responses to the open-ended question regarding other comments on the model and its utility in training were overwhelmingly positive, especially from novices. Studies suggest that fun and enjoyment contribute to educational motivation and help with retention of knowledge (22, 23).

During use of the model, we encountered several areas for potential improvement. The model would occasionally count continuous contacts (“infinity contact”). Some participants noted that the beeping associated with infinity contacts increased their stress level and made it harder to assess whether they were actually contacting the sides of the tube. From a study perspective, this also means that contacts may have been underestimated during infinity contacts if additional contact occurred during the time the pre-infinity number was recorded and the counter reset. It is possible that the infinity beeps could be eliminated if the rheostat of the sensitivity knob was more precise or if there was a designed refractory period after each beep.

Although the model was relatively portable and durable, some of the participants inadvertently removed the number or fixed stars during the simulation. These were easily replaced, but would add to stress and inefficiency for the trainee. We could consider having the stars be loosely magnetized to the side of the tube so that they are removable but less likely to be inadvertently deflected off.

With respect to validity evidence, modern validity theory purports all validity evidence is a type of “construct validity (24, 25).” Whereas older validity frameworks tended to focus primarily on the accuracy and reproducibility of results, modern validity frameworks focus on the appropriateness of the inferences made about a set of results. Kane’s framework for construct validity is largely considered by most education and psychology scholars to be the most appropriate framework for evaluating validity evidence (25). Kane’s framework for construct validity suggests there are four primary elements of interest: scoring inferences; generalization inferences; extrapolation inferences; and implication inferences (24–26).

In the present study, limited validity evidence that speaks to three of the four aforementioned elements are discernible. More specifically, the content representation, construction and implementation of the standardized instrument speaks to the appropriateness of scoring inferences; the decision accuracy, decision consistency and adequate group samples of experts and novices speaks to appropriate generalization inferences; and the instrument’s ability to provide a challenging and effective training model that reflects authentic clinical performance and aids in the development of hand control and indirect hand-eye coordination skills speaks to appropriate extrapolation inferences. We present no evidence that speaks to implication inferences, as we did not examine the impact of the model on a training program or investigate the potential impact on patient outcomes.

There are several additional limitations of this study as an exploration of the use of the model as a pedagogic tool. We did not have an objective test of hand-eye-screen coordination and thus relied upon user comments of the usefulness of the model to train these skills.

In summary, this manuscript describes the development and initial evaluation of a low-fidelity rigid rhinoscopy training model. Our data support the further and continued development and use of this model. This low fidelity rhinoscopy model is inexpensive and relatively easy to make. Questionnaire results from experts supported the utility of the model to train rigid rhinoscopy skills (e.g., hand-eye coordination, instrument handling). Novices reported that the model was enjoyable to use, was appropriately challenging, and would improve their rhinoscopy skills. As such, this model appears to be a good basis for further refinement of a low-fidelity rigid rhinoscopy training model that could be used in further studies to assess its impact on performance in the workplace. A more complex model may improve the training of all veterinary rhinoscopists, enhancing the standard of care for patients undergoing rhinoscopy.

## Data Availability

The original contributions presented in the study are included in the article/Supplementary material, further inquiries can be directed to the corresponding author.
