# Supplementary material for: Development and initial evaluation of a rigid rhinoscopy model as a pedagogic tool in veterinary medicine
Source: Front Vet Sci. 2024 Sep 10;11:1356026. doi: 10.3389/fvets.2024.1356026 (PMC11420988; doi:10.3389/fvets.2024.1356026)
Supplement: Supplementary file 1 [file Data_Sheet_1.PDF]

Preliminary Final Design: Force Matrix

The Force Matrix was the best option in regards to the design matrix, Dr. Pritchard's wishes, and the team's capabilities. The next iteration of the design has three simple tubes (Figure 6.1) in which a smaller hexagonal elastic tube would fit inside. The outer tubes would be threaded to make it modular, which makes it easy to remove the tubes, make repairs, and clean. The elastic tube would be wrapped with a layer of conductive fabric, followed by velostat and copper. The three tubes would then be connected to an arduino that would supply power and read voltage drops when a rigid probe made contact with the inner elastic tube.

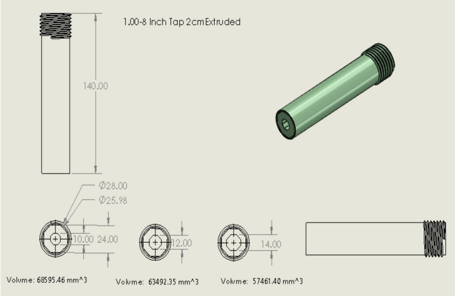

Figure 6.1. SolidWorks sketch of a nose tube

The overall size of the model (Figures 6.2 and 6.3) is 160mm X 80mm X 115mm(LxWxH). The spaces that house the tubes are 28mm in diameter, with the rear being threaded to hold the tubes in place. In order to have increasing difficulty, the tubes were scaled having a larger diameter in the bottom tube and were decreased by 2mm for each of the next two tubes. A potentiometer was also added to the control box that could increase the sensitivity of the sensor.

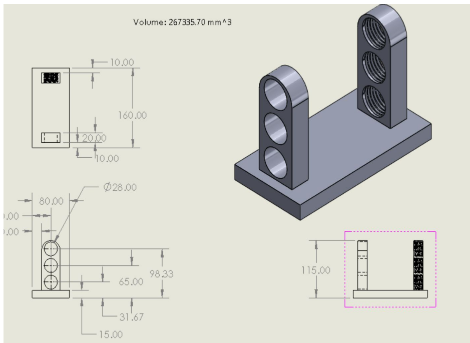

Figure 6.2. SolidWorks sketch of the Frame

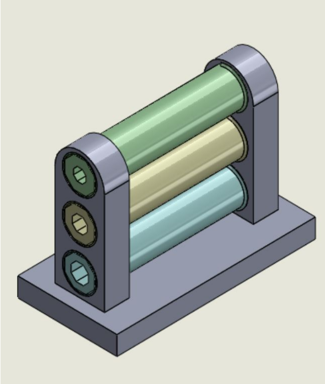

Figure 6.3. Solidworks sketch of frame with the nose tubes in place

This sensor will function via a simple arduino and breadboard (Figure 6.4). When an endoscope applies enough pressure to the inner wall of the tubes, the current of the circuit will increase due to a decrease in the velostat's resistance. This will be counted as an error.

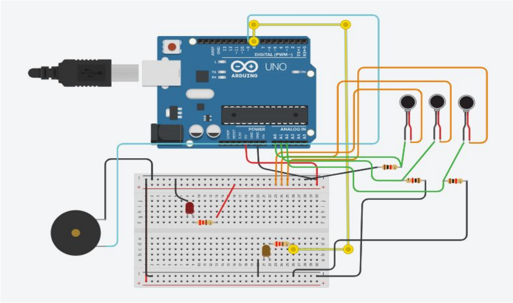

Figure 6.4. Arduino and breadboard schematic showing part of the final circuit

Fabrication

The 3D printing process was used for fabricating the frame and physical structure of the model. SolidWorks was used to model the frame, three rigid tubes, three flexible tubes, a handle, and an alar fold. Different materials were used to print these parts: PLA for the frame, rigid tubes, and the handle, and elastic resin for the flexible tubes and alar fold. A wooden dowel wrapped with sandpaper was used with a drill to sand down the tube inserts on the frame to achieve the proper, smooth fit. Epoxy was used to glue the handle to the frame. Threaded brass inserts were melted into the plastic frame. A locking mechanism for the tubes was created by putting screws in these inserts.

The sensors were fabricated by wrapping one layer of conductive fabric around the inner flexible tube, followed by a layer of velostat. The velostat layer overlapped itself slightly so the two conductors could not touch directly. The rigid copper foil layer was made to tightly fit against the inner walls of the outer PLA tube. The sensor assembly was then friction fit into the PLA tubes. A piece of thin clear plastic was glued across the back of this assembly to hold the sensor together. Room was left on either side of this cross bar for the conductive fabric and copper foil tabs which protrude in order to connect the sensor to the arduino.

The electronic part of the model was fabricated by soldering the circuit (Figure 7.1) onto a breadboard-layout perfboard. Headers were soldered to connect the wires to the correct arduino and LCD screen pins. Then, the arduino code (Appendix B) was uploaded to the controller. 2-pin buttons were also soldered on and screwed into the electronics box for the purposes of starting and stopping the timer, zeroing the sensors before a session, and resetting the system. Potentiometers were used for adjusting sensitivity and backlight brightness on the LCD screen. More detailed instructions for fabricating the Rigid Rhinoscopy Training Model can be found in Appendix C.

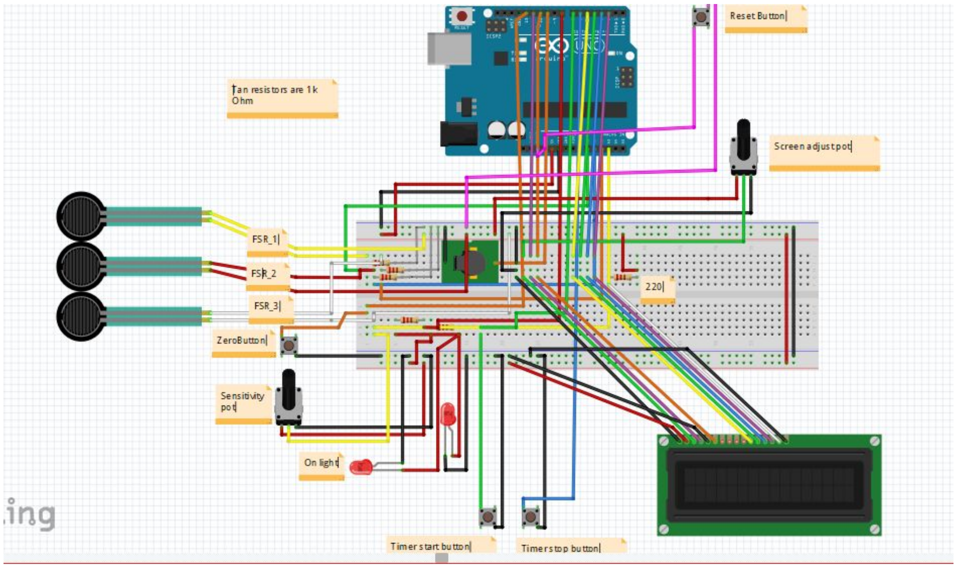

Figure 7.1. Perfboard and Arduino schematic

A pelican case was also bought and molded to fit the shape of the final product (Figure 7.2). This provides a safe place for storage and protection during shipping. The budget for this project was $2,000 and the final cost of the product was $416.41.

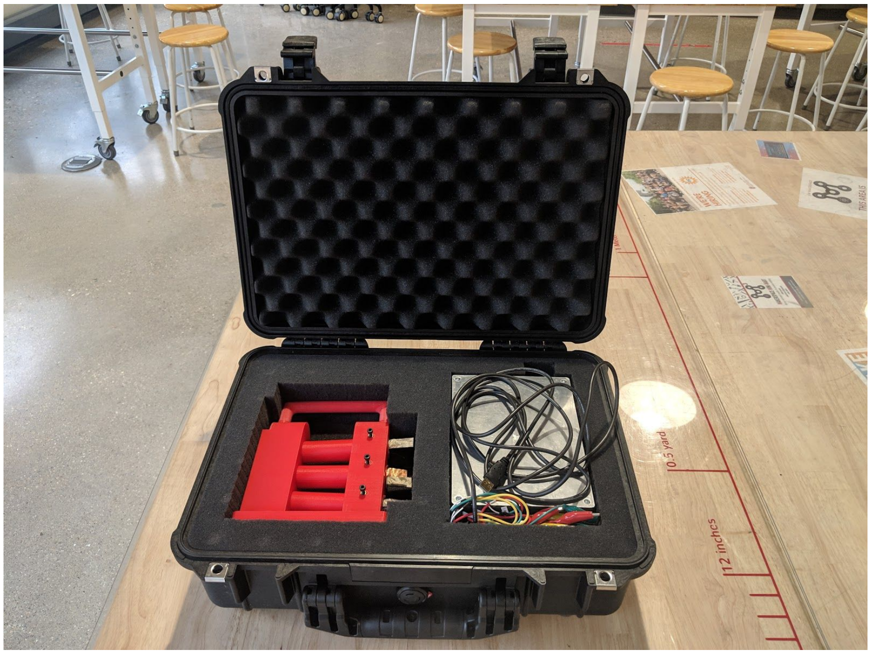

Figure 7.2. Final shipping packaging for the training model.
