# Supplementary material for: Development and initial evaluation of a rigid rhinoscopy model as a pedagogic tool in veterinary medicine
Source: Front Vet Sci. 2024 Sep 10;11:1356026. doi: 10.3389/fvets.2024.1356026 (PMC11420988; doi:10.3389/fvets.2024.1356026)
Supplement: Supplementary file 3 [file Data_Sheet_3.DOCX]

**Rigid rhinoscopy simulator – post-participant questionnaire** (Experts)

| Please mark **ONE** box per question | **Strongly Disagree** | **Disagree** | **Agree** | **Strongly Agree** |
| --- | --- | --- | --- | --- |
| 1. The use of this model is an **effective way to train skills** applicable to rigid rhinoscopy |  |  |  |  |
| 1. This model provides training appropriate for the performance of rigid rhinoscopy **with respect to hand control** |  |  |  |  |
| 1. This model provides training appropriate for the performance of rigid rhinoscopy **with respect to indirect hand-eye coordination** |  |  |  |  |
| 1. The use of this model is an **appropriately challenging way to promote** **growth of skills** applicable to rigid rhinoscopy |  |  |  |  |
| 1. The use of this model is an **enjoyable way to train skills** applicable to rigid rhinoscopy |  |  |  |  |
| 1. The use of this model is a **frustrating way to train skills** applicable to rigid rhinoscopy |  |  |  |  |

1. What aspects of this model did you find particularly applicable to the training of novices in canine rhinoscopy?

________________________________________________________________________________________________________________________________________________________________________________________________________________________

1. What aspects of this model would you change if you had the opportunity and why?

________________________________________________________________________________________________________________________________________________________________________________________________________________________

1. Please provide any additional feedback on the model or exercises. Feel free to continue any comments on the back of this sheet.

________________________________________________________________________
